# Purkait’s triangle revisited: role in sex and ancestry estimation

**DOI:** 10.1080/20961790.2021.1963396

**Published:** 2022-02-14

**Authors:** MennattAllah Hassan Attia, Mohamed Hassan Attia, Yasmin Tarek Farghaly, Bassam Ahmed El-Sayed Abulnoor, Sotiris K. Manolis, Ruma Purkait, Douglas H. Ubelaker

**Affiliations:** a Forensic Medicine & Clinical Toxicology Department, Faculty of Medicine, Alexandria University, Alexandria, Egypt; b Institute for Intelligent Systems Research and Innovation, Deakin University, Victoria, Australia; c Biomedical Engineering, Medical Research Institute, Alexandria University, Alexandria, Egypt; d Diagnostic Radiology, Faculty of Medicine, Alexandria University, Alexandria, Egypt; e Fixed Prosthodontics, Faculty of Dentistry, Fayoum University, Faiyum, Egypt; f Animal & Human Physiology, Faculty of Biology, National & Kapodistrian University of Athens, Athens, Greece; g Department of Anthropology, University of Allahabad, Allahabad, India; h Department of Anthropology, Smithsonian Institution, Washington, DC, USA

**Keywords:** Forensic sciences, forensic anthropology, Purkait’s triangle, fragmentary femora, ancestry estimation, random forest modelling, international forensic investigations

## Abstract

Identification of unknown remains recovered from marine and terrestrial locations is a significant humanitarian problem. This investigation proposes a simple method applicable to fragmentary femora for a more refined level of ancestry and/or sex estimation. To that end, we re-examined Purkait’s triangle which involves three inter-landmark distances between the traction epiphyses and the articular rim of femoral head. A large sample (*n* = 584) from geographically diverse (Egyptian, Indian and Greek) populations was compiled. Additionally, shape (*n* = 3) and trigonometrically derived variables and ratios (*n* = 9 variables) were employed to detect any geographically-clustered morphological differences between these populations. Random forest modelling (RFM) and linear discriminant function analysis (LDA) were employed to create classification models in instances where sex was known or unknown. The sample was apportioned into training and test sets with a ratio 70/30. The classification accuracies were evaluated by means of *k* fold cross-validation procedure. In sex estimation, RFM showed similar performance to LDA. However, RFM outperformed LDA in ancestry estimation. Ancestry estimation was satisfactory in the Indian and Egyptian samples albeit the Greek sample was problematic. The Greek samples presented greater morphological overlap with the Indian sample due to high within-group variation. Test samples were accurately assigned to their ancestral category when sex was known. Generally, higher classification accuracies in the validation sample were obtained in the sex-specific model of females than in males. Using RFM and the linear variables, the overall accuracy reached 83% which is distributed as 95%, 71% and 86% for the Egyptian, Indian and Greek females, respectively; whereas in males, the overall accuracy is 72% and is distributed as 58%, 87% and 50% for the Egyptian, Indian and Greek males, respectively. Classification accuracies were also calculated per group in the test data using the 12 derived variables. For the females, the accuracies using the medians model was comparable to the linear model whereas in males the angles model outperformed the linear model for each group but with similar overall accuracy. The classification rates of male specific ancestry were 82%, 78% and 56% for the Egyptian, Indian and Greek males, respectively. In conclusion, Purkait’s triangle has potential utility in ancestry and sex estimation albeit it is not possible to separate all groups successfully with the same efficiency. Intrapopulation variation may impact the accuracy of assigned group membership in forensic contexts.

Key pointsPurkait’s method is a possible ancestry group indicator applicable to fragmentary femora.Random forest model surpassed linear discriminant function analysis in multi-group ancestry classification.Ancestry is more accurately assessed in females than males.The intertrochanteric distance is the most important feature in discrimination of sex whereas in ancestry it was the head to lesser trochanter distance.Sex differences override ancestry due to the tendency of misclassification into same sex but different group rather than the opposite sex of the same ancestry.

## Introduction

Sex and geographic ancestry are fundamental components of the biological profile of decedents that should be established in order to expedite the settlement of justice [1–3]. Ancestry is defined as the geographic region of origin of an individual which is not interchangeable with race [3, 4]. Ancestry estimation in forensic anthropology generally aims to classify an unknown individual to the most likely geographic origin using statistical classification models [1]. Demonstrably valid methods may aid in the positive identification of unknown remains encountered in challenging forensic circumstances such as cases of mutilation, commingling or dispersal where only single skeletal element or fragments could be analyzed [2, 3].

The impetus for ancestry assessment in research and practice is halted by the variations in global practice of forensic anthropology because of political issues, demographic composition of the country as well as regional medico-legal demands [5, 6]. Nevertheless, it is a typical requirement of international forensic casework in natural or manmade disasters and humanitarian settings as a consequence of the complex systems of interrelations [5, 7, 8]. The domestic and international laws and crime prevention are the building blocks for establishing security, human rights and peace in the world [9]. The conducted studies have been employed to distinguish between the broad socially constructed ancestral group in North America [2], South Africa [10] and Balkan region [11] with limited capacity to differentiate between only two to four groups within the same geographical area which poses a significant problem for the other populations in the world [12].

In the Middle East and North African (MENA) region, Egyptian labour migration is the principal migration pattern to Arab region and Europe. India has the largest diasporas in the world. The US, UAE and Saudi Arabia host the Indian migrants in addition to other gulf states since the “oil boom” in the 1970s [13, 14]. Over the last few years the number of migrants residing in the Gulf Cooperation Council (GCC) countries has increased considerably where non-nationals outnumber national citizens in some countries [15]. By examining the composition of Gulf labour market, Egyptians and Indians represent the major constituents of the demographic, economic and cultural fabric of the GCC countries [16]. Subsequently, the traditional forensic casework may involve unknown remains/victims from both populations.

On the other hand, Greece is the fourth country in the European Union as a destination for refugees and asylum seekers from the Arab region according to the Missing Migrants Project [17]. The humanitarian tragedy due to a loss of human life at sea is particularly linked to the irregular migration surge through maritime routes such as those crossing the Mediterranean. Between 1988 and 2013 a reported 14 309 people died in an attempt for migration using low quality vessels [18]. Whilst the majority of maritime fatalities are related to migration, other recent prominent incidents resulting in a large number of individuals died in the sea for example the crash of Egypt Air flight 804 killed 66 passengers in the Mediterranean in 2016 [19].

For possible identification of missing persons from a closed list, investigators must have complete information on the age at death, sex, ancestry, stature and time since death in order to meet the demands of the legal process [20]. Ancestry estimation is a difficult undertaking in forensic anthropology owing to the complexity of the concept and confusion in interpretation [21, 22]. The crania provide unique information for positive scientific identification in anthropological analysis [23]. The post-cranial elements also present abundant anatomical features useful for identification [24]. The traditional research focus on the skull particularly the midfacial region, over the post-cranial, bones [1, 3, 6, 23–25]. By contrast, sex estimation is a straightforward procedure using almost any skeletal element [25]. Thus, the pursuit of alternative methods using the appendicular skeletal elements is beneficial not only to provide more corroborative evidence to cranial findings but to make specimens available for use when some cranial information are not recovered [26, 27].

Post-cranial metric methods of estimating ancestry have found the most success with the lower limb bones and pelvic girdle, especially the femur [28, 29]. The design of the proximal femur, that is, the head, neck and its internal architecture supports the mechanical stress and strain that influence bone remodelling and density (Wolff’s law) [30]. A number of studies have shown marked ancestral differences in the shape of the proximal femur and in at least one trait of the distal femur-intercondylar notch height [31]. Other aspects of the femur, such as the femoral neck length [32], platymeria [11, 12, 33], and anterior femoral curvature [34], have shown differences among the major geographic populations.

Early models used in sex and ancestry estimation involved classical multivariate analysis techniques such as linear discriminant function analysis in FORDISC 3.0. [35, 36] and logistic regression models [36]. Recently, researchers have used a novel algorithm called random forest modelling (RFM) in both craniometric and morphoscopic approaches of ancestry estimation [27, 37]. Over the last decade, machine learning algorithms have brought a whirlwind of new insights to human variation. Moreover, they surpassed traditional classification methods in anthropological research, even when all of their statistical assumptions are met [37, 38].

In 2005, a method of sex estimation was developed by Purkait [39] that involved three inter-landmark distances taken from traction epiphyses where muscles inserted and connected to the most lateral point of the head of femur at articular rim. The performance of the lengths of the triangle varies among populations as regards to the allocation accuracies and degree of sexual dimorphism [40–42]. Inspired by the illustration created by Anastopoulou et al. [40] and linking this with the different classification rates obtained from previous validation studies of the method, we hypothesized that such changes might to some extent underpin the size and shape differences of the proximal femur observed among the three studied populations: Egyptian, Indian and Greek.

Hitherto, no research has been carried out to determine if the anatomical variation in proximal femur as captured by Purkait’s triangle can be used to reconstruct ancestry of unknown individuals. To that end, the construction of a large identified sample from geographically distant populations in conjunction with an intelligent statistical modelling technique by means of RFM would quantify the morphometric characteristics of the femur in each group and also likely indicate the accuracy of the ancestry estimates. We also compared the classification ability of RFM with models generated through linear discriminant function analysis (LDA). If the overall classification accuracy is demonstrated to remain higher than chance alone, then Purkait’s triangle can be used as a criterion for estimating the ancestry of the remains, thus greatly contributing to forensic studies on identity reconstruction.

## Materials and methods

### Reference samples

In order to evaluate the applicability of Purkait’s triangle for sex and/or ancestry estimation, we used matched femoral metric data of identified individuals from three geographically distant populations, namely the Egyptian, Indian and Greek samples. Overall, 584 individuals were analyzed, with a total of 352 males and 232 females representing a sex ratio of 1.5:1. Table 1 depicts the sample size, characteristics and demographic data.

**Table 1.** The description of reference samples according to sample size, source and nature of metric data.

Population	Origin/source of data	Total sample	Male:female	Mean of age (min–max) (year)	Socioeconomic status and social attributes	Nature of data
Egyptian	Hospital patients attending the radiology department	101	40:61	46.6 (19–77)	Middle-to-low class contemporary residents of Alexandria Governorate	3D VR models of living individuals from a database of abdominal/pelvic CT images
Indian [39]	Forensic cases from the Skeletal Collection housed at the Medico-Legal Institute at Bhopal	280	200:80	N/A	Middle class residents of Central India	Skeletal sample
Greek [40, 43]	Department of Animal and Human Physiology, Faculty of Biology, University of Athens	203	112:91	55.1 (19–99)	Middle-to-low class residents of Athens	Skeletal sample

3D: three dimensional; VR: volume rendering; CT: computerized tomography.

### Metric data collection

The femoral Digital Imaging and Communications in Medicine (DICOM) datasets were obtained from a 64-slice multi-slice Computerized tomography (CT)scanner manufactured by Siemens Somatom^®^ Perspective (Siemens Medical Solutions USA, Malvern, PA, USA) and based on slice thickness of 1.5 mm slices. Three dimensional (3D) reconstruction of proximal femur was performed using the RadiAnt DICOM Viewer (v.5.0.2 64-bit; Medixant, Poznań, Poland) for Windows [44]. The different aspects of the dataset were interactively explored in the 3D VR (volume rendering) tool window, rotated 180° to visualize the posterior aspect of the femur in order to obtain three inter-landmark distances of Purkait’s triangle following the description in the original paper of Purkait [39]. The acquisition of measurements was conducted by a single observer (the third author) to eliminate inter-observer errors. The measurements of the Indian [39] and Greek [40] samples were previously collected manually using Dial and Mitutoyo digital callipers of 0.01 mm accuracy for the published original studies, respectively.

**Figure 1.**
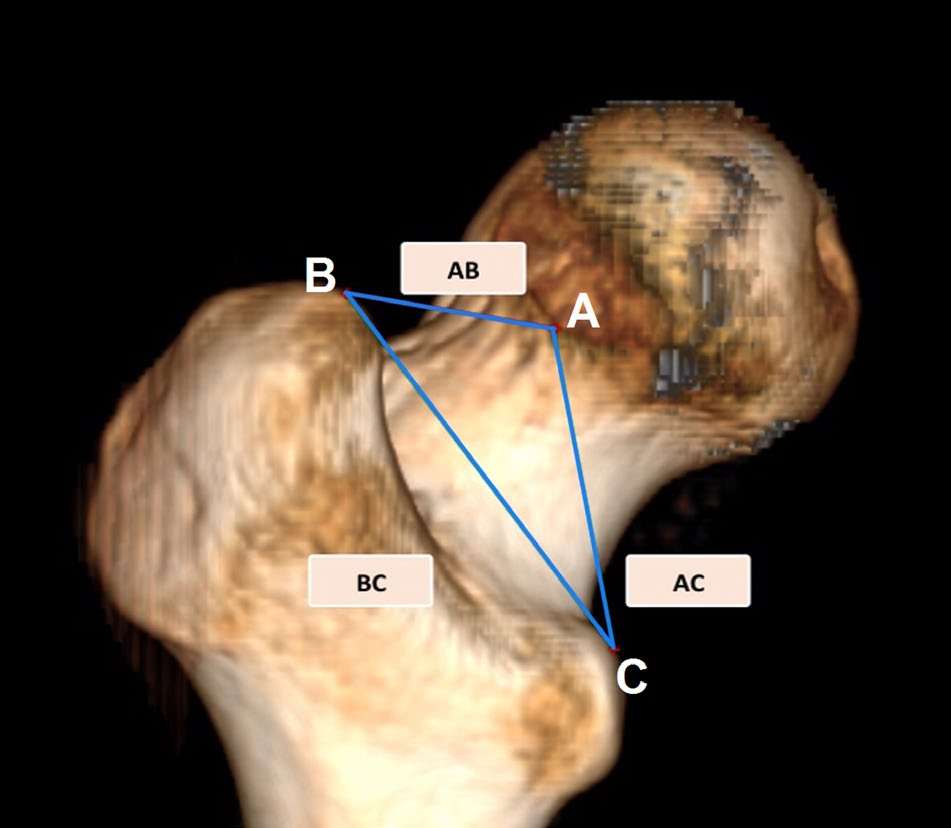
The three linear measurements of Purkait’s triangle labelled on the posterior aspect of 3D volume rendered CT image of left Egyptian femur. The point B is located on the greater trochanter and it is projecting most medially. Point C is the highest point projecting most medially on the lesser trochanter, while point A was located on the articular margin of head dipping most laterally.

Measurements were exclusively taken on the left proximal femur with the exception of the Indian sample which were taken from both sides and only one side of each was included [39]. We excluded cases with history of bilateral pelvic/femoral fractures, deformity, previous hip operation, age-related diseases and bone tumours. Figure 1 represents a graphical representation of the three measurements.

### Evaluation of the technical error of measurements

Since the current study is retrospective in nature, a unified strategy in error quantification was not possible. Intra-observer error was evaluated for the measurements obtained from the Egyptian virtually reconstructed femur models using the technical error of measurement (TEM), relative TEM (rTEM) and the coefficient of reliability (*R*) [45]. Prior to data collection, a subsample of 10 femora were randomly selected representing approximately 10% of the total sample size (the recommended range is 10%–20%) [46]. Each variable was measured twice, and each set of observations was made 1 month apart using the same method of measurement.

### Evaluation of the degree sexual dimorphism and inter-population comparisons

Descriptive statistics of the three measurements were computed in order to provide a characterization of the reference samples analysed in the current study. One-way ANOVA was performed to evaluate the relationships of each variable in Purkait’s triangle with sex and ancestry. Variables were tested for normality (Kolmogorov–Smirnov test) for the two groups (males and females) in each population sample. Separate ANOVA were run on male and female individuals of each population due to sex differences in size. Tukey honestly significant difference (HSD) *post hoc* tests were used to determine which ancestral groups differed from one another by each variable in terms of the size and patterns of sexual dimorphism. The coefficient of variation (CV) was computed using the formula [(SD/*x* × 100]. It was implemented in order to compare the normally distributed data with respect to their variability [39, 47].

### Sex and ancestry estimation

#### The variables

The three inter-landmark distances and other variables (*n* = 9) including the angles, the angle to measurement ratios and the medians have also been considered. The angles were defined by the linear measurements where Angle A, B and C are opposite to BC, AC and AB. The angles were calculated using the Law of Cosines [48]. Ratios between the linear measurement and angles were used because a larger individual may have a larger triangle with no measurable change in the angles of the triangle. Therefore, the angle data relative to the triangle lengths may be more efficient predictors of sex and/or ancestry. The ratios were employed following Albanese et al. [48] as:

- The ratio of Angle A divided by the length AB multiplied by 100.
- The ratio of Angle B divided by the length BC multiplied by 100.
- The ratio of Angle C divided by the length AC multiplied by 100.

Medians of triangle were calculated following the Apollonius’ theorem [49]. These variables were pooled and employed in a single model to evaluate their efficiency in improving the accuracy of the sex and ancestry classification tasks. Medians consider the distances between the femoral head, greater and lesser trochanters in relation to each other.

Three additional shape variables ABsh, ACsh, BCsh were computed using Darroch and Mosimann (1985) method [50]. The shape variables are the three original variables divided by the geometric mean for each case. Working with the shape variables control for size-related shape differences, therefore sexes can be combined for ancestry estimation.

#### Modelling techniques

Each group of the three linear and the newly derived trigonometric variables of Purkait’s triangle were modelled for sex and/or ancestry estimation using the LDA and RFM included in the Scikit-learn [51] in Python (version 0.22) [52].

LDA calculates the multivariate distance between groups using the group-specific means and a pooled within-group variance–covariance matrix (VCM), then classifies the observation discriminant score (expressed as a linear combination of a set of three variables from Purkait’s triangle or the pooled variables) within one of the several groups under experiment depending on whether the score is higher or lower than the threshold. LDA requires fulfilment of three statistical assumptions: (i) the explanatory variables within each class should have a multivariate normal distribution; (ii) equal VCMs of the groups; (iii) low correlation between explanatory variables [38].

RFM is trained using bootstrap aggregation (Bagging) which is the process of repeatedly testing randomly drawn samples from the original training data (bootstraps), repeating the process and refining the model over several trees and then aggregating the models learned on each bootstrap. A subset of the variables is selected randomly and whichever variable gives the best split is used to split the node iteratively. After creating all the decision trees in the forest, out-of-bag (OOB) error is the average prediction error for each training sample calculated using predictions from trees that do not contain this particular sample in their respective bootstrap sample. This allows the model to be fit and validated while being trained. OOB error is an estimate of generalization (prediction) error of unseen data. Bagging technique has methods for balancing errors in training datasets where classes are imbalanced. Further, it overcomes variance (overfitting) by training multiple decision trees on different subspace of the feature space at the cost of slightly increased bias (i.e. underfitting due to systematic under or over prediction of the target groups) [37].

Two variable importance measures (VIMs) or rankings are calculated during the analysis: mean decrease in accuracy (MDA) and mean decrease in impurity (MDI) [53]. In both cases, the higher a variable is on the visualization chart, the more important it is determined to be. MDA is based upon the mean decrease of accuracy in predictions on the OOB samples when a given variable is permuted (randomly changed) in the OOB samples when passed down the trees of the model. It is a direct measure of the impact of each feature on the accuracy of the model. MDI is defined as the total decrease in node impurity averaged over all trees of the ensemble calculated for each variable separately and the features are ranked according to this measure. Nevertheless, calculation of the MDA is the most advanced way to measure importance, while the MDI is more like a proxy because it is influenced by the correlation between variables and it depends on the data [54] as any of these correlated variables can be used as the predictor reducing the importance of others.

The sklearn.random_projection module was implemented as a computationally simple and efficient way to reduce the dimensionality of the data in order to visualize them in a 2D plot. The dimensions and distribution of random projections matrices are controlled so as to preserve the pairwise distances between any two data points in the dataset in the original space. Thus random projection is a conventional approximation technique for biological distance based studies [55].

#### Experiments design, tuning and assessment of performance

The present study builds methodologically upon Liebenberg et al. [10], which runs separate analyses to classify the remains according to (i) sex only within the three populations pooled, (ii) ancestry only by geographic level with sexes pooled, (iii) sex and ancestry simultaneously, and (iv) sex-specific ancestry where the recorded sex allowed the partitioning of the pooled dataset into male and female groups to train the models separately to gauge the effect of prior knowledge of sex on the correct identification of ancestry [10]. The training/test sets design was employed in which each model was developed from 70% of the sample and tested on the remaining 30%. The number of individuals in each experiment varied by the model according to the intended classification task. In the multi-class situation, the One-Vs-All decomposition scheme was implemented to distinguish between a single class (positives) and the remaining ones (negatives) [56].

## Results

### Evaluation of the measurement reproducibility and reliability

Table 2 shows that AB measurement had the highest magnitude of measurement error in relation to the size of the measurement (rTEM = 3.38%) with an intermediate margin of error as demonstrated by the value of TEM (0.97 mm). This was followed by the AC measurement, which had an rTEM of 2.77% and a relatively higher TEM = 1.10 mm. Both measurements exhibited coefficient of reliability (*R*) equal to 0.95. The lowest error was achieved in the BC measurement where the calculated rTEM, TEM and *R* were 1.37%, 0.81 mm and 0.97, respectively. These results are within the recommended range of human error [57, 58].

**Table 2.** Results obtained for the absolute technical error of measurement (TEM), the relative technical error of measurement (rTEM) and the coefficient of reliability (*R*) showing intra-observer reproducibility for the subsample *n* = 10 (two repeats). Refer Figure 1 for the definitions of AB, BC, and AC.

Measurement	TEM (mm)	rTEM (%)	*R*
AB	0.97	3.38	0.95
BC	0.81	1.37	0.97
AC	1.10	2.77	0.95

### Evaluation of the sexual dimorphism and inter-population comparisons of the reference samples

The descriptive statistics of each variable by geographic origin and in the pooled sample are summarized in Table 3. All measurements are statistically significantly different between the males and females with the exception of AB in the Greek sample. The differences between samples in the dimensions of Purkait’s triangle within each sex and pooled sex groups were examined using one-way ANOVA as shown in Table 4. Statistically significant differences in the three dimensions among the three groups were found in each sex and pooled sample. *Post hoc* comparisons showed that significant differences consistently exist in AC diameter among all three population groups in both males and females.

**Table 3.** Summary statistics (in mm), coefficient of variation (CV), sexual dimorphism index (SDI) (reported in %) and ANOVA on *df* = 1 for three measurements by sex. Refer Figure 1 for the definitions of AB, BC, and AC.

Measurement	Group	Sex	Mean	SD	Min	Max	CV	SDI	*P*	*F*
AB	Egyptian	Females	26.80	3.71	19.00	40.00	13.8	8.99	0.003*	9.03
		Males	29.45	5.13	14.00	41.00	17.4			
	Greek	Females	29.69	5.59	18.52	59.83	18.8	4.21	0.4775	0.51
		Males	30.25	5.56	16.67	45.93	18.4			
	Indian	Females	30.15	4.47	21.8	42.15	14.8	9.02	<0.0001*	19.70
		Males	33.14	5.31	18.00	48.00	16.02			
	Pooled	Females	29.09	4.95	18.52	59.83	17.01	8.52	<0.0001*	36.04
		Males	31.80	5.58	14.00	48	17.55			
BC	Egyptian	Females	55.11	4.34	40.00	65.00	7.87	11.18	<0.0001*	48.41
		Males	62.05	5.66	50.00	76.00	9.12			
	Greek	Females	53.26	4.79	42.13	67.19	8.99	10.46	<0.0001*	74.55
		Males	59.38	5.20	47.14	71.73	8.76			
	Indian	Females	51.74	5.51	41.00	63.15	10.65	13.84	<0.0001*	159.10
		Males	60.05	4.75	49.6	79.00	7.91			
	Pooled	Females	53.22	5.09	40.00	67.19	9.56	11.39	<0.0001*	254.77
		Males	60.06	5.05	47.14	79.00	8.41			
AC	Egyptian	Females	37.90	4.86	27.00	57.00	12.82	9.44	0.0003*	14.12
		Males	41.85	5.60	29.00	58.00	13.38			
	Greek	Females	45.06	5.44	24.89	59.34	12.07	11.62	<0.0001*	55.32
		Males	51.55	6.71	36.66	71.71	13.02			
	Indian	Females	40.14	4.39	27.60	47.90	10.94	18.92	<0.0001*	174.34
		Males	49.51	5.7	36.10	65.15	11.51			
	Pooled	Females	41.48	5.77	24.89	59.34	13.91	15.84	<0.0001*	213.68
		Males	49.29	6.64	29.00	71.71	13.47			

Note: *P < 0.05, indicating significant difference.

**Table 4.** ANOVA for each of the three measurements of Purkait’s triangle by ancestry in each sex separately and the pooled sex sample.

Measurement		Between female groups		Between male groups		Between pooled sex groups	
		*P*	*F*	*P*	*F*	*P*	*F*
AB	All	<0.0001*	9.69	<0.0001*	14.67	<0.0001	29.44
	GRK–EGY	0.0009*		0.6978		0.0024*	
	IND–EGY	0.0002*		0.0003*		<0.0001*	
	IND–GRK	0.8060		0.00002*		0.00001*	
BC	All	0.0004*	8.08	0.0150*	4.21	0.0052*	5.31
	GRK–EGY	0.0625		0.0111*		0.1835	
	IND–EGY	0.0002*		0.0557		0.0065*	
	IND–GRK	0.1125		0.4932		0.1378	
AC	All	<0.0001*	42.73	<0.0001*	38.42	<0.0001*	66.45
	GRK–EGY	<0.0001*		<0.0001*		<0.0001*	
	IND–EGY	0.0225*		<0.0001*		<0.0001*	
	IND–GRK	<0.0001*		0.0122*		0.0094*	

GRK: Greeks; EGY: Egyptians; IND: Indians.

* *P* <0 .05, indicating significant difference.

Moreover, the Indian males have a significantly longer AB and shorter AC distances than those of the Greeks. Thus, the superior point on the greater trochanter was high relative to the head. Further, Greek males have the highest within-sex variation, especially in the AB diameter whereas Indian males scored the lowest CV in all measurements (Table 3). Indian females have a significantly shorter AC distance than Greeks. Although the BC and AB measurements are not significantly different, the CV of the BC and AB were the highest and the second highest values among Indian females. Given that AB and AC dimensions are the shortest in Egyptian females and males, this means that the neck length of Egyptian females and males is on average shorter than that of the Indians and Greeks as measured from the head border dipping most laterally (Tables 3 and 4). These differences are indicating the existence of morphological differences of proximal femur among the three samples.

### Performance of linear discriminant function vs. RFM in sex and ancestry estimation

**Figure 2.**
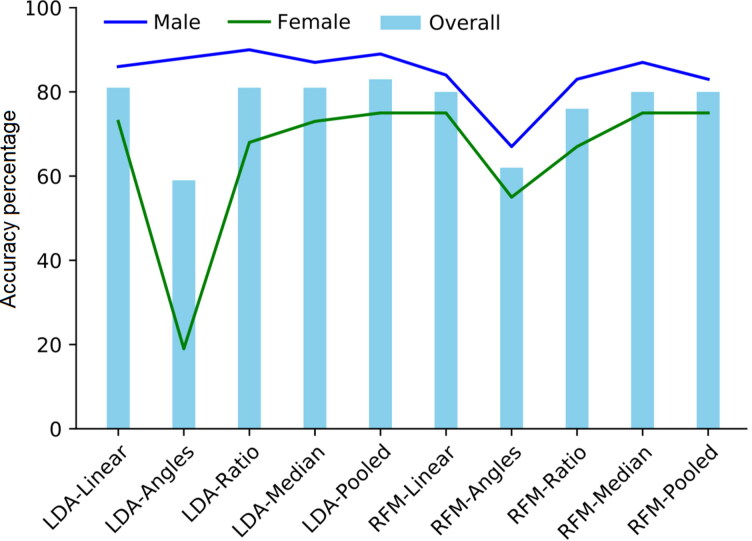
Comparison between the different variables models using linear discriminant function analysis (LDA) and random forest modelling (RFM) on test data in sex only. LDA outperformed RFM in all the models except the angles model. A drop in the classification accuracy of females in favour of male accurate classification by LDA using the angles model is observed in this plot. RFM demonstrated less drop in both males and females which refers to the ability of RFM to classify difficult samples.

Supplementary Tables S1–S6 provide the classification accuracies for training and test samples, by the individual models. Supplementary Table S1 depicts the classification accuracies of sex only models using the three variables of Purkait’s triangle. Results in the test sample showed that the performance of RFM was generally comparable to the LDA model (the overall accuracies were 80% and 81%, respectively), with a higher misclassification rate of females than males by both models. Some of the newly generated variables showed different performance from the linear ones (Figure 2). In LDA and RFM, the medians model performance was more or less similar to the linear variables models with slight improvement in male classification accuracy. The females classification accuracies using the angles models in both LDA and RFM greatly declined in favour of more accurate male classification rates. It is not surprising that the ratio of angle to linear variables models showed an intermediate performance between the linear and angles models. For the pooled variables LDA model, the sex allocation accuracies improved by 3% and 2% in males and females, respectively. In the RFM pooled variables models, a similar performance to the linear model in the overall accuracy and female sex allocation was obtained with a trivial decrease in male sex accuracy by 1%.

**Figure 3.**
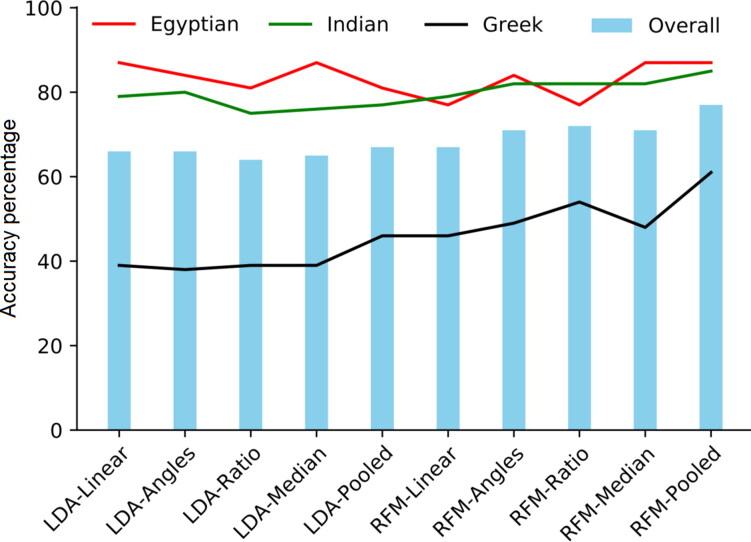
Comparison between the different variables models using linear discriminant function analysis (LDA) and random forest modelling (RFM) on test data in ancestry only experiment. RFM outperformed LDA in all the models. An improvement in the overall as well as per group classification accuracies can be visualized in the plot by RFM pooled variables model. The pooled variables LDA model is nearly similar to the linear variables RFM model.

However, LDA and RFM for ancestry estimation did not show equal performance. In Supplementary Table S2, the ancestry only models based on Random forest outperformed LDA with accurate classification of 95% *versus* 65% of the training samples to the appropriate ancestral group whereas in the test sample, comparable overall accuracy was obtained being 67% *versus* 66% of the test samples. Looking at each group separately in the test sample, the Egyptians were accurately classified 87% (LDA) and 77% (RFM) of the time, while the Indian were accurately classified 79% of the time by both classifiers. Only 39% of the Greeks were correctly classified by LDA but RFM improved the accuracy to 46%. The models including the newly computed variables, however, resulted in 81%–87% correct group membership *versus* 77%–87% for the Egyptians using LDA and RFM, respectively and for the Indians 75%–80% *versus* 82%–85% using LDA and RFM, respectively. For the Greek sample, 38%–39% *versus* 49%–54% were correctly classified using LDA and RFM, respectively. Surprisingly, the pooled variables model showed 15% higher accuracy for the Greek sample using RFM which outperformed the LDA pooled variables for the same sample. Generally, the pooled variables models improved the classification accuracies over the linear models using LDA and RFM (Figure 3). In Supplementary Table S3, the shape variables models resulted in 65% (LDA) and 62% (RFM) overall classification accuracy in the test sample albeit the Greek sample showed the lowest accurate classification in both classifiers being 31% *versus* 35% for the LDA and RFM, respectively.

**Figure 4.**
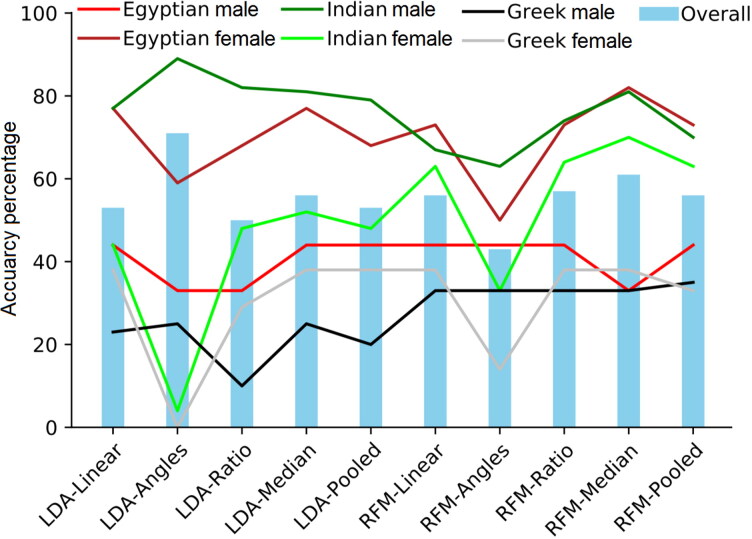
Comparison between the different variables models using linear discriminant function analysis (LDA) and random forest modelling (RFM) on test data in sex and ancestry simultaneously experiment. RFM outperformed LDA in all the models except the angles model. A drop in the female groups classification accuracies can be visualized in the plot by the angles models in both LDA and RFM.

In Supplementary Table S4, the six-way classification models of sex and ancestry simultaneously classified only 53% and 56% of the test sample using the LDA and RFM, respectively. While an accuracy of 53%–56% is considered low, in the six-way classification, chance is calculated as 16.7% (using six groups and assuming equal prior probabilities). Hence, accuracy is approximately 40% greater than chance alone. The best performance by LDA and RFM was encountered in the Egyptian females and Indian males followed by Indian females and Egyptian males. Greek males showed the lowest accuracies with 23% and 33%, using LDA and RFM, respectively. Generally, RFM achieved higher accuracies in the overall accuracy and each subgroup but with slightly lower Egyptian females and Indian males accuracies than LDA leading to more balanced classification accuracies without bias towards certain group. Classification accuracies were also calculated per group for both classifiers in the test data using the new variables. A drop in the females allocation accuracy regardless their ancestral origin was noted by both classifiers using the angles and subsequently the ratio models. The medians and to lesser extent the pooled variables models achieved comparable or slightly better performance than the linear variables models using both LDA and RFM (Figure 4).

**Figure 5.**
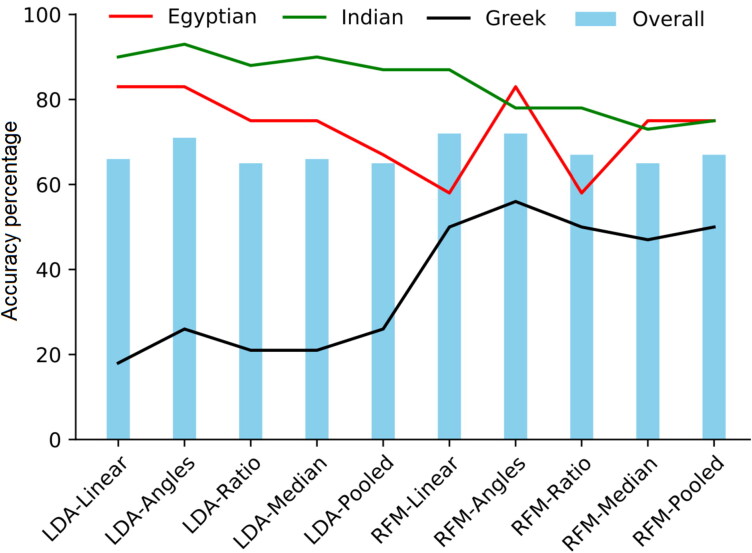
Comparison between the different variables models using linear discriminant function analysis (LDA) and random forest modelling (RFM) on test data in the females ancestry experiment. RFM outperformed LDA in all the models. An improvement in the Indians and Egyptians group classification accuracies can be visualized in the plot by RFM medians model. Generally, the LDA model showed obvious bias with higher misclassification rates in the Indian females.

**Figure 6.**
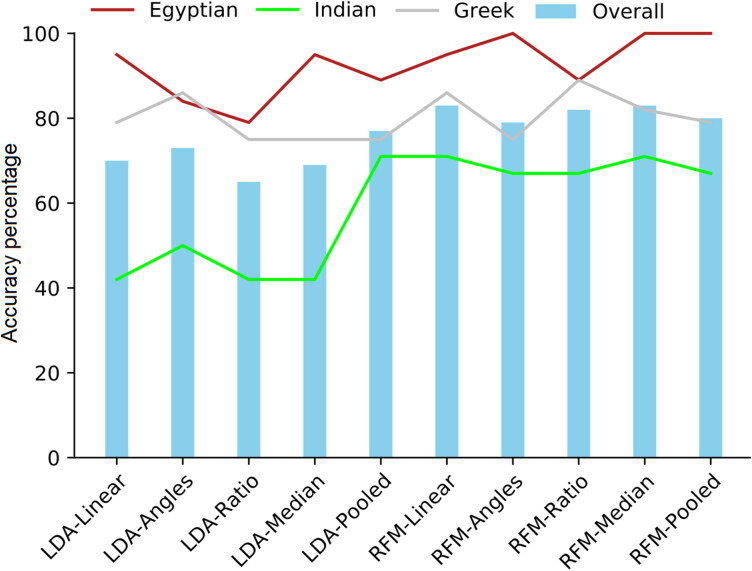
Comparison between the different variables models using linear discriminant function analysis (LDA) and random forest modelling (RFM) on test data in the males ancestry experiment. RFM outperformed LDA in all the models. A marked improvement in the Greeks and Egyptians group classification accuracies can be visualized in the plot by RFM angels variables model. Generally, the LDA model showed obvious bias to Egyptians and Indians with higher misclassification rates in the Greeks.

By considering ancestry estimation when the sex is not pooled, Supplementary Tables S5 and S6 demonstrate that the RFM outperformed LDA in the overall classification accuracies as well as the classification rate for the Greek males and Indian females resulting in a more consistent classification accuracies among the groups in each sex. In general, higher classification accuracies were achieved in the known female sex model than in the known male sex model. While these two models require sex determination prior to ancestry estimation, the known sex model could be useful in circumstances where there is a higher likelihood of finding certain sex remains and in closed crime scenes contexts or where other traits indicate one sex either male or female, but further assessment of ancestry is needed. By comparing the performance of the new variables to the linear variables in the females experiments, the LDA pooled variables model achieved higher classification accuracy rates than the linear variables model, whereas RFM demonstrated more or less similar performance using the medians to the linear models (Figure 5). In male ancestry estimation, both LDA and RFM angles models outperformed the linear variables model. RFM achieved the highest Greek male classification with accuracy of 56% *versus* 26% using the LDA model (Figure 6).

**Table 5.** Cross-cultural classification rate of previously published full formula (three linear variables).

Population	Males (%)	Females (%)	Overall (%)
Indian [28] on Egyptians	50	93.3	66.34
Greek [29] on Egyptians	62.5	86.88	77.22
Indian [28] on Greek [29]	84.54	63.53	74.04

**Figure 7.**
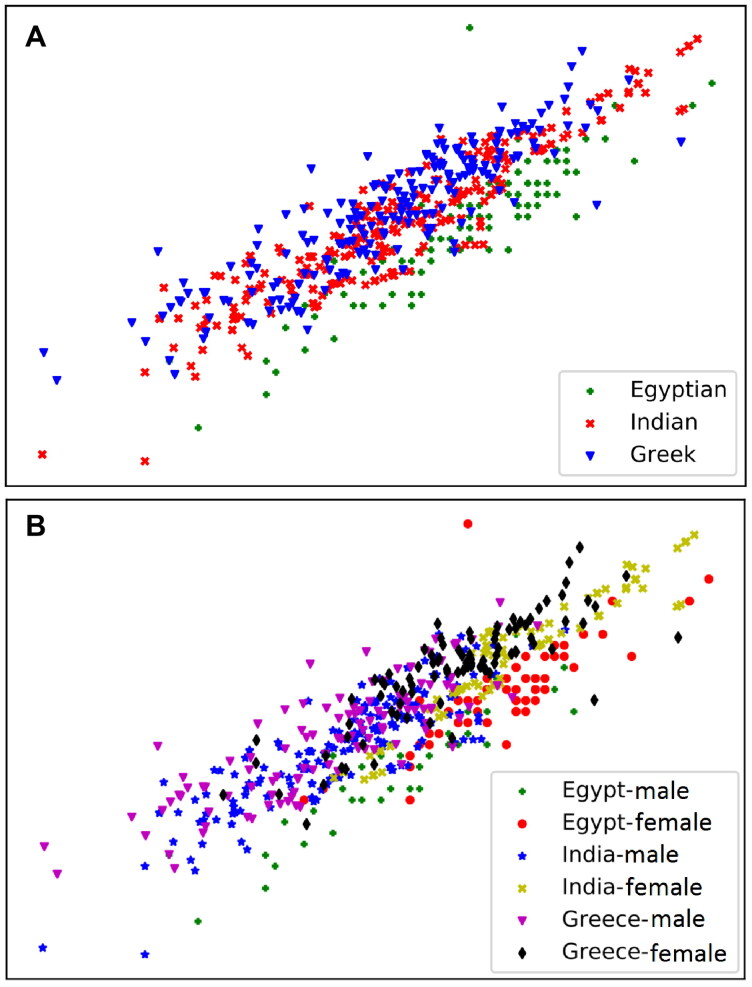
Plot of random projection of features for dimensional reduction demonstrating group variation and overlap among the Egyptian, Indian and Greek populations when using the three measurements of Purkait’s triangle. (A) Ancestry only cohort showed considerable overlap between the Indian and Greek populations with better separation of the Egyptians. (B) In the sex-ancestry cohorts, clear clustering of data by sex and better separation of the populations among the females samples.

Figure 7 is labelled according to (i) ancestry, (ii) sex and ancestry to illustrate the extent of overlap among the geographical ancestry groups. As seen in Figure 7A, a substantial overlap between the Indian and Greek groups is revealed, explaining why the Greek individuals were more prone to misclassification into the Indian group. The Egyptian sample is clearly separated from the two other samples. As such, the highest classification rates were obtained by LDA and RFM models in the Egyptian group in the ancestry only and ancestry in females experiments.

In the sex-ancestry cohorts, the plot in Figure 7B reveals the considerable overlap between the males in the Indian and Greek populations explaining why Greek individuals are more likely to misclassify for the Indian group. This indicates the greater overlap among the populations by ancestry than by sex. Therefore, individuals of the same sex are more likely to misclassify into a different ancestry group rather than the opposite sex of the same ancestry group. However, Egyptian males and Greek females are still more likely to misclassify to the opposite sex rather than misclassifying according to ancestry (into the incorrect sex). The confusion matrices in the Supplementary Figures S1–S5 reveal these patterns. Visualizations of the variable importance measures in RFM of each experiment are included as Supplementary Figures S6–S11.

## Discussion

In the present study, contemporaneous Egyptian population metric data were compiled to construct a virtual skeletal database using 3D volume rendered computed tomography (3D-CT) technology while overcoming the cultural and legal constraints, mitigating the time-consuming nature and tediousness of skeletal maceration or the need for physical storage space [59–61]. One notable contribution of this work is emphasizing on the importance of collaborative research and data sharing which are invaluable for the development of new databases. The availability of such virtual databases are paramount to account for modern human skeletal morphological variations, particularly for the identification of human remains in modern forensic cases. Hence, the goal of the current project was the distillation of any presumed patterns of morphological dissimilarity in these broadly sampled populations.

### Sex estimation

In the sex classification task, RFM achieved better sex-specific and overall accuracy than the application of the population specific function on each population (Table 5). Generally, the overall accuracy obtained from the generalized models are within the range of 81%–87% reported in the published population specific studies [39–42, 48]. A relatively high sex bias towards male sex allocation in the sex only model was observed. Since the mean values for the female measurements of the BC in the Egyptian, AC in the Greek, and AB in the Indian populations variables were slightly larger than the mean values of the variables in the pooled data, we would expect a decreased accuracy in females because there will be, on average, more females larger in size and thus increase the overlap with smaller males. Together with the higher coefficient of variation of BC measurement (most important feature) in the pooled female sample (9.56%) than male samples (8.41%), this may lead to overestimation of the measurements in a considerable number of females in each population [62].

Brown and colleagues [41] found that BC diameter was determined to be the most important variable in the pooled ancestry sample from the Terry collection, a finding consistent with our results using RFM. Similarly, Anastopoulou et al. [40], Purkait [39] and Djorojevic et al. [42] achieved higher accuracy using the BC variable than the other variables. All these studies [39–42] also reported higher classification accuracy when the three variables were employed simultaneously in models.

While the BC dimension reflects upper body weight transmission and muscular development as insertion regions of muscles, the AC diameter reflects the femur neck length and position of the lesser trochanter relative to the femur head [40]. Arguably, several clinical orthopaedic and bioanthropological studies [30, 32] suggested the presence of both sex and population differences in the femur neck axis length. These differences stem from the fact that the femur neck axis length is correlated with the general femoral strength [32]. Thus, it is not surprising that the AC dimension was selected as the most important variable in ancestry estimation and the second most important variable in sex estimation. Furthermore, it is obvious that there are different size variables operating in the discrimination of sex and ancestry [26].

### Ancestry estimation

In the ancestry classification tasks, several trends can be observed. First, the Indian group demonstrated better resolution from the Egyptian and Greek groups using Sex Pooled and Sex Unknown attempts. Surprisingly, 54% of femora from the Greek sample were classified into the Indian group using the Sex Unknown and 50% for the Greek males and less than 14% for the Greek females using the Sex Known information (see the confusion matrices in the electronic supplemental materials and Supplementary Tables S4 and S5). The Egyptian sample showed overall better classification accuracies than the Greek sample for both males and females. The Egyptian females and males have a shorter femur neck on average than individuals of both sexes in all the other populations that have been studied.

On the other hand, the lower classification rate for the Greek male sample is likely due to the substantial overlap existing between the Indian and Greek males but not in their female counterparts where the pattern is reversed due to the high intrapopulation variation in the femoral morphology in the Greek males and Indian females (Figure 7B and Tables 3 and 4). Nevertheless, Ousley and associates [63] and Witherspoon et al. [64] acknowledged the presence of large amounts of within-group variation leading to modest differences between populations, however, accurate group allocation is still possible.

Anastopoulou et al. [40] presented a schematic comparison of the Purkait’s triangles of the right femora for each sex in four populations: Greek, Indian, European-American and African-American populations. They observed that while the triangle for Greek males does not differ significantly in terms of size and shape from the other populations, the triangle of Greek females is considerably different in both aspects. This may explain the successful ancestry estimation among the Greek females better than males.

In our study, the shape variables adequately separated the Egyptians and Indians from the other ancestral groups, however, in Greeks the classification accuracies fall below the linear variables. The shape of the proximal end of femur was quantified as a vector of ratios: each of the three measurements were divided by a standard size variable (geometric mean). A standard size variable quantifies the overall size of the bone and scales linearly in relation to the raw measurements [50]. Thus, it preserves the aspect ratio without removing the allometric effects of size variation. This method also identified cases with similar shape but have different sizes [65–67] (indicated by the high misclassification rate of the Greeks to the Indians group being 69% and 66% for the LDA and RFM models, respectively). Since the possibility of genetic admixture can be excluded owing to the geographical separation, the results may also reflect the nature of the Athens collection (Greek) samples being from the middle to low socioeconomic classes with variable nutritional status [43, 68]. Taking into consideration that points B and C are placed in the area of muscle attachments and upper body weight transmission, the differences in anatomy of this region in terms of size and shape might then be a reflection of social conditions. The distance between traction epiphyses (BC) in the Indian and Greek (males and females) individuals was non-significant, indicating that they were exposed to similar access to strenuous forms of labour [69]. Moreover, the shape variable BCsh was the most important feature in the ancestry only experiment. This may explain the drop in the classification accuracy of the Greek sample. Nevertheless, marked improvement in the overall classification rates with 15% higher accuracy for the Greeks were observed in the ancestry only experiment using the pooled variables model (shape variables were not included).

Second, the calculation of separate estimates of the ancestral groups for males and females in this study had a positive impact on the classification accuracies. This observation is in concert with the recommendation by Komar [70, p. 163] to separate the ancestral categories by sex as a method of increasing reliability. Sex accounts for a major proportion of the variation among groups and ancestry accounts for most of that remaining [71] as shown in Figure 7B. Taking the effects of sexual dimorphism into account through the use of Sex Known models for ancestry analyses achieves better allocation accuracies for Greek males. Moreover, these models require sex to be determined prior to estimating ancestry, which may not be possible in all cases. The most surprising of these results can be seen in the degree of similarity between Indian and Greek male individuals is diminished using the angles models due to elimination of the size effect and the demonstration of the differences in the position of the femoral head to the greater and lesser trochanters (see Section “Evaluation of the sexual dimorphism and inter-population comparisons of the reference samples”). Angles are computed as ratio using only two sides of triangle and this procedure introduces an additional one degree of freedom that allows for more variability. Thus, angles enhanced the classification accuracies. On the other hand, in the sex and ancestry experiment, the Egyptians, Indians and Greek females were misclassified to the opposite sex of the same population rather than other ancestry group using the angles model. This might be relevant to the observation that sexual differences in the femoral neck–shaft angle (NSA) are minor and inconsistent [72]. The same finding was evident in the sex only experiment where the classification accuracies of females dropped using this model. In the Ancestry in females experiment, the medians model was comparable to the linear measurements model achieving the best accuracies in all of the three populations because size may be a significant discriminatory factor in females.

Third, the ancestry of females is more accurately assessed than males using our method. The accuracy of group classification in females was more balanced in each population and higher than in their male counterparts with the exception of the Indian males (source of imbalance in the male group). This observation is consistent with ancestry estimation in several settings using postcranial bones [10, 29]. Using LDA, Holliday and Falsetti [29] achieved 100% accurate ancestry classification in females *versus* 87% of male training samples. Liebenberg et al. [10] reported higher allocation accuracy for black females (70%) than in males (67%) and in coloured females (80%) than in males (73%) whereas in white South African males and females both were classified equally (93%).

### Performance of LDA versus RFM

The model accuracy using LDA was comparable to RFM in the binary classification of sex. Male and female groups are nearly linearly separable using a combination of the three explanatory variables [73]. Notwithstanding, RFMs proved to provide better performance than LDA in ancestry estimation (multi-group classification). LDA has long been considered as the standard classification statistic for ancestry [3]. In the present work, RFM demonstrated lower classification bias of the difficult groups, that is, Greek males and Indian females than LDA in the different variables (Figures 2–6). Hence, the predictive performance is reliable due to efficient estimation of the feature importance and test error. The results for the test sample were not as high as the training sample, however, this reflects the coarse resolution of accuracy estimates because small validation samples suffer from larger variance [38] rather than an error in the training procedure (i.e. overfitting) as evidenced from Figures 2–5. Since the three population samples were not proportionally represented in the sample, this may have had an effect on the classification accuracies. A larger validation sample would improve classification [38]. Moreover, in multi-group classification, certain groups will often demonstrate higher classification rates than other groups, with the tendency of some groups to be misclassified into specific groups due to similarities to each other than to other groups, so maximizing correct classification rates may be a challenging task [74].

The results also showed that none of the new variables was found to be significant for sex assessment. This is in agreement with a previous Spanish study where the accuracy of sex estimation did not increase using the angles, areas and ratios between these variables [42]. The model of pooled variables does not improve the accuracy over the traditional linear inter-landmark distances. Interestingly, the pool of variables in LDA approached the accuracy by RFM. This indicates that RFM learn from the complexities in the underlying pattern of the training data and the influence of a predictor variable directly corresponds to its discriminatory power, interaction with other variables, and position in the tree of the ensemble [37, 53, 75].

While there are no studies exploring the utility of populations differences in Purkait’s triangle in the proximal femur for ancestry estimation, a handful of previous studies have explored the shape and size differences in raw measurements and ratios of femur for ancestry estimation [31–35, 76]. In 2015, Shirley et al. [76] explored the shape differences between the African and European Americans using LDA and principal component analysis (PCA). They found that the stepwise procedure selected the lesser trochanter-head centre distance among the top 10 significant variables for discrimination between both groups using femur and/or tibia. This particular variable is readily comparable to the AC measurement in Purkait’s triangle without the need of complicated procedures to determine the virtual head centre. Moreover, the PCA demonstrated that the greater trochanter area is the most significant site for differences between both groups which is one of the components of the Purkait’s triangle.

The results of ancestry estimates in the previous studies vary with the type and number of variables employed, available skeletal elements, as well as the population studied. For example, the utilization of the femur neck axis length (FNAL) alone achieved 46.2% in a six-way analysis of sex and ancestry in American European, African, and Native individuals with complete failure in allocation of American African females. A male sex-specific three- and two-way analysis provided overall accurate classification of 57.1% and 53.2%, respectively [32]. These classification accuracies are lower than the rates of classification achieved by our models.

Furthermore, the findings herein indicate comparable or even better results to the overall accuracy (60%) reported by Lienberberg [10] in a three-way analysis of ancestry regardless of sex in the South African population and the 63% reported by Spradley [24] in the male specific ancestry analysis of North American European and African individuals *versus* Hispanic male individuals using seven variables namely femur maximum length, femur epicondylar breadth, femur maximum diameter of head, femur anterior–posterior subtrochanteric diameter, femur transverse subtrochanteric diameter, femur anterior–posterior diameter at midshaft, femur circumference at midshaft.

Using multiple postcranial bones in the LDA, better overall results were obtained being 79% and 85% accurate classification in the North American [24] and South African [10] populations, respectively. Dibennardo and Taylor [26] used a combination of 15 pelvic and femoral measurements from White and Black Americans in the Terry Collection and achieved a high predictive accuracy of 95% for sex and ancestry simultaneously. These measurements capture the morphological differences of femur between the African and European Americans as well as the proportional differences in lower limb length to torso length between both groups as related to ecogeographical patterns in body form [29]. Notwithstanding, the inclusion of two variables (i.e. the maximum femoral length and iliac height) demonstrated an overall accuracy of 87% for ancestry prediction regardless of sex. Similarly, Holliday and Falsetti [29] collected the maximum length of the femur in addition to other long bone lengths, trunk height and bi-iliac breadth then devised a sex-specific LDA. The training sample consisted of African Americans and European Americans from the Terry Collection and was tested on a modern sample of forensic anthropology cases. They achieved an overall cross-validation classification rate of 93.5% for American Black and White males and females however, the test sample did not perform well, and it was attributed to secular change. FORDISC 3.0 provided an overall classification rate of 92.2% for modern American Black and White individuals using all postcranial bone lengths or heights available [24, 36]. Notwithstanding, the complete skeleton in good condition for the acquisition of these measurements is rarely encountered in real casework due to taphonomic factors [32].

Whilst the findings of these aforementioned studies cannot be directly compared with the results of this study, it is clear that sex and ancestry differences in the morphology of posterior aspect of femur can be discerned. Given the previously reported populations differences in proximal femur morphology, it should come as no surprise that the reported classification rates to the appropriate population group are more than chance but are based solidly on the intelligent classifier (i.e. RFM) employed in the current investigation. This patterning is — in part — thought to be the result of genetic drift as well as natural selection and adaptation to highly varied microenvironments in worldwide populations [24, 63, 77].

Purkait’s triangle method combats several issues encountered in ancestry estimation, for example, the measurements are typically reproducible, and relatively simple, with few number of variables can be collected. Purkait’s triangle captures an approximation of femur neck length and angle simultaneously. There is a paucity of research on the femoral NSA due to problems and uncertainties regarding the best method for determining the NSA. Moreover, the complex anatomical aspects of the proximal femur make measurement difficult, including the lack of reliable landmarks and the irregularities of the neck contours, which makes the definition of the neck axis ambiguous [48, 78]. These findings strengthen the value of the triangle in ancestry estimation using simple, flexible and rapid technique rather than the complicated methods of Geometric Morphometric techniques because these measurements can be collected directly from the dry skeletal elements using callipers and do not require the availability of adjuvant radiological imaging facilities [76].

A great utility pertinent to its applicability in forensic case scenarios where partial remains are recovered with intact proximal portion of femur. On land, post-cranial bones may be less affected by animal scavenging and other post-mortem factors [48, 79]. The proximal end of femur is more likely to be recovered in various terrestrial scenarios [32, 48, 57]. On the other hand, the recovery and identification efforts of human remains from the sea are affected by the poorly understood marine taphonomy effects [18]. In the sea, floating of the human remains weaken the soft tissue connecting the major joints leading to disarticulation of the appendages from distal to proximal; on the upper limbs first followed by the lower limb through current and successive wave action. On the lower limbs loss of the feet at the ankle joint is followed by the knee joint while the hip joint is usually preserved in connection with the trunk. The disarticulation pattern of the cranium and mandible parallels that of the upper limb where the mandible is lost with hands, and the cranium disarticulates with the forearms. The sunken remains show more severe scavenging and skeletonization than those floating [18]. Therefore, proper recovery and analysis of the entire human remains evidence are paramount to positive identification [80].

The comprehensive morphological information presented in this paper may be useful in the event that the cranium is unavailable for examination or in instances in which Supplementary Information is needed. Even better, the use of multiple elements approach offers the potential for combined probabilities and likelihood that should enhance the identification effort in order to achieve the required levels of reliability for forensic applications. Thus, the proposed method might be considered for use in forensic cases involving these three populations while practicing some caution with Greek males. The difficulty in Greek (European) male allocation supports the need for a more refined level of ancestry estimation to fully account for human variation at the population-level [77]. It must be emphasized though that the samples may not be representative of the whole region of Greece and India, which creates the need for larger validation studies.

One should also note that Greek individuals are accurately separated from the Egyptians which may refer to the applicability in tracking deaths of migrants through the Mediterranean routes. Further, migration in the Arab region is multi-faceted and varies in the scope between countries. Conflicts in the region have led to over 16 million refugees, mainly in Iraq, Libya, Somalia, the Sudan, the Syrian Arab Republic and Yemen. Syrian refugees represented one-third of them since 2011 [14]. Large numbers of migrants are transiting through the Arab region and across the Mediterranean [81]. Recognizing the diverse and complex migration, refugees and displacement dynamics in the MENA regions poses legal and social challenges that should be addressed for establishing peace and justice in these regions.

Finally, to what extent the overall body mass/size could underpin the intrapopulation variation of the shape of the proximal femur as opposed to inter-population differences remains to be established. Therefore, evaluation of populations from different backgrounds such as Arab migrants and other nationalities needs more attention in future studies to be able to assess the general usability of the method for more accurate and precise ancestry estimates.

## Conclusion

The use of standardized procedures for investigations of the dead by forensic scientists and investigative agencies involved in the management of dead migrants is paramount to ensure a dignified management of the tragedic incidents. The results presented here are the first, comprehensive analysis of variation in the morphology of the proximal femur as captured by Purkait’s measurements. The core elements to the proposed methodology include analysis of geographically distant groups for insightful study of the diversification of modern humans, applying robust modelling techniques to reveal the subtle differences among the pooled sample, and the demonstration of the differences between the most important variables selected for each classification task. However, the distinctions among geographically distant populations may be blurred as a result of significant intra-population variation which may adversely impact the correct allocation into appropriate ancestry groups. Undetected and/or undocumented intra- and inter-group variations beyond the historical three-group model have significant bearing on forensic anthropological casework.

## Authors’ contributions

MennattAllah Hassan Attia conceived of the study, and participated in its design and coordination and drafted the manuscript. Yasmin Tarek Farghaly carried out the Egyptian data collection and DICOM file processing, participated in the study design and helped to draft the manuscript; Mohamed Hassan Attia and Bassam Ahmed El-Sayed Abulnoor participated in the study design and performed the statistical analysis in Python and R programming languages, respectively. Sotiris K. Manolis and Ruma Purkait carried out Greek and Indian data collection, respectively. They provided resources and supervised writing the first draft of the manuscript. They participated in reviewing and editing of the manuscript. Douglas H. Ubelaker provided resources and supervised writing the first draft of the manuscript. He participated in reviewing and editing of the manuscript; All authors contributed to the final text and approved it.

## Compliance with ethical standard

This research study was conducted retrospectively from radiological database obtained for clinical purposes with institutional approvals. The study protocol has been approved by the Research Ethics Committee of faculty of medicine, Alexandria University, Egypt. (IRB NO.: 00012098 - FWA NO.: 00018699, Serial NO. 0304532).

## References

[1] Spradley MK. Metric methods for the biological profile in forensic anthropology: sex, ancestry, and stature. Acad Forensic Pathol. 2016;6:391–399. 31239914 10.23907/2016.040 PMC6474557

[2] Konigsberg LW, Algee-Hewitt BF, Steadman DW. Estimation and evidence in forensic anthropology: sex and race. Am J Phys Anthropol. 2009;139:77–90. 19226642 10.1002/ajpa.20934

[3] Dunn RR, Spiros MC, Kamnikar KR, et al. Ancestry estimation in forensic anthropology: a review. Wiley Interdiscip Rev: Forensic Sci. 2020;2:e1369.

[4] SWGANTH, Scientific Working Group for Forensic Anthropology Ancestry assessment. [cited 2021 Apr 15]. Available from: http://swganth.org/products–drafts.html

[5] Ubelaker DH. The global practice of forensic science. Chichester (UK): Wiley-Blackwell; 2015.

[6] Chrysostomou P, Thompson TJ. Anthropology: ancestry assessment. In: Payne-James J, Byard RW, editors. Encyclopedia of forensic and legal medicine. 2nd ed. Oxford (UK): Elsevier; 2016. p. 162–168.

[7] Kimmerle EH, Jantz RL, Konigsberg LW, et al. Skeletal estimation and identification in American and East European populations. J Forensic Sci. 2008;53:524–532. 18471195 10.1111/j.1556-4029.2008.00708.x

[8] Hefner JT, Byrnes JF. Globalization, transnationalism, and the analytical feasibility of ancestry estimation. In: Garvin HM, Langley NR, editors. Case studies in forensic anthropology: bonified skeletons. Boca Raton (FL): CRC Press; 2019.

[9] Cattaneo C, Binz MT, Penados L, et al. The forgotten tragedy of unidentified dead in the Mediterranean. Forensic Sci Int. 2015;250:e1–e2. 25770023 10.1016/j.forsciint.2015.02.007

[10] Liebenberg L, Krüger GC, L’Abbé EN, et al. Postcraniometric sex and ancestry estimation in South Africa: a validation study. Int J Legal Med. 2019;133:289–296. 29797281 10.1007/s00414-018-1865-x

[11] McIlvaine BK, Schepartz LA. Femoral subtrochanteric shape variation in Albania: implications for use in forensic applications. HOMO. 2015;66:79–89. 25500529 10.1016/j.jchb.2014.09.004

[12] Tallman SD, Winburn AP. Forensic applicability of femur subtrochanteric shape to ancestry assessment in Thai and White American males. J Forensic Sci. 2015;60:1283–1289. 25845441 10.1111/1556-4029.12775

[13] United Nations. International Migration 2019. New York (NY): Department of Economic and Social Affairs. [Report ST/ESA/SER.A/438]. Available from: https://www.un.org/en/development/desa/population/migration/publications/migrationreport/docs/InternationalMigration2019_Report.pdf

[14] United Nations. 2017 Situation report on international migration: migration in the Arab region and the 2030 Agenda for sustainable development international migration. Beirut Lebanon (Lebanon): ESCWA; 2018. Available from: http://digitallibrary.un.org/record/3797358/files/E_ESCWA_SDD_2017_1-EN.pdf

[15] International Labour Organization (ILO). Labour migration: facts and figures. Available from: https://www.ilo.org/beirut/areasofwork/labour-migration/lang–en/index.htm

[16] Hamza S. Migrant labor in the Arabian Gulf: a case study of Dubai, UAE. Pursuit The J Undergra Res Univ Tennessee. 2015;6:10. Available from: http://trace.tennessee.edu/pursuit/vol6/iss1/10

[17] Missing Migrants Project. International Organization of Migration (IOM). [cited 2021 Mar 15]. Available from: http://missingmigrants.iom.int/

[18] Ellingham ST, Perich P, Tidball-Binz M. The fate of human remains in a maritime context and feasibility for forensic humanitarian action to assist in their recovery and identification. Forensic Sci Int. 2017;279:229–234. 28934682 10.1016/j.forsciint.2017.07.039

[19] Hradecky S. Crash: Egypt A320 over Mediterranean on May 19th 2016, aircraft found crashed, ACARS messages indicate fire on board. The Aviation Herald [Internet]. 2016. May 19 [cited 2016 May 21]. Available from: http://avherald.com/h?article=4987fb09

[20] Kranioti EF, Garcia-Donas JG, Karell MA, et al. Metric variation of the tibia in the Mediterranean: implications in forensic identification. Forensic Sci Int. 2019;299:223–228. 31055136 10.1016/j.forsciint.2019.03.044

[21] Sierp I, Henneberg M. Can ancestry be consistently determined from the skeleton? Anthropol Rev. 2015;78:21–31.

[22] Ousley S, Jantz RL, Hefner JT. From Blumenbach to Howells: the slow, painful emergence of theory through forensic race estimation. In: Boyd Jr CC, Boyd DC, editors. Forensic anthropology: theoretical framework and scientific basis. New York (NY): Wiley, 2018. p. 67–97.

[23] Rhine S. Skeletal criteria for racial attribution. Ann Anthropol Pract. 2012;13:54–67.

[24] Spradley K. Metric ancestry estimation from the postcranial skeleton. In: Berg GE, Ta’ala SC, editors. Biological affinity in forensic identification of human skeletal remains: beyond Black and White. Boca Raton (FL): CRC Press; 2014. p. 83–94.

[25] Curran BK. The application of measures of midfacial projection for racial classification. In: Gill GW, Rhine S, editors. Skeletal attribution of race. Albuquerque (NM): University of New Mexico; 1990. p. 55–57.

[26] Dibennardo R, Taylor JV. Multiple discriminant function analysis of sex and race in the postcranial skeleton. Am J Phys Anthropol. 1983;61:305–314. 6614145 10.1002/ajpa.1330610305

[27] Spiros MC, Hefner JT. Ancestry estimation using cranial and postcranial macromorphoscopic traits. J Forensic Sci. 2020;65:921–929. 10.1111/1556-4029.14231 31725188

[28] İşcan MY. Cotton TS. Osteometric assessment of racial affinity from multiple sites in the postcranial skeleton. In: Gill GW, Rhine S, editors. Skeletal attribution of race. Albuquerque (NM): University of New Mexico; 1990. p. 83–90.

[29] Holliday TW, Falsetti AB. A new method for discriminating African-American from European-American skeletons using postcranial osteometrics reflective of body shape. J Forensic Sci. 1999;44:926–930. 10486943

[30] Wheatley BP. An evaluation of sex and body weight determination from the proximal femur using DXA technology and its potential for forensic anthropology. Forensic Sci Int. 2005;147:141–145. 15567618 10.1016/j.forsciint.2004.09.076

[31] Baker SJ, Gill GW, Kieffer DA. Race & sex determination from the intercondylar notch of the distal femur. In: Gill GW, Rhine S, editors. Skeletal attribution of race: methods for forensic anthropology. Albuquerque (NM): University of New Mexico; 1990. p. 91–95.

[32] Meeusen RA, Christensen AM, Hefner JT. The use of femoral neck axis length to estimate sex and ancestry. J Forensic Sci. 2015;60:1300–1304. 26258403 10.1111/1556-4029.12820

[33] Wescott D, Srikanta D. Testing assumptions of the Gilbert and Gill method for assessing ancestry using the femur subtrochanteric shape. HOMO. 2008;59:347–363. 19007928 10.1016/j.jchb.2008.05.002

[34] Ballard ME, Trudell MB. Anterior femoral curvature revisited: race assessment from the femur. J Forensic Sci. 1999;44:700–707. 10432602

[35] Ousley SD, Jantz RL. FORDISC 3 and statistical methods for estimating sex and ancestry. In: Dirkmaat DC, editor. A companion to forensic anthropology. Oxford (UK): Blackwell Publishing Ltd.; 2012. p. 311–329.

[36] Jantz RL, Ousley SD. FORDISC 3.0: personal computer forensic discriminant functions. Knoxville (TN): University of Tennessee; 2005.

[37] Navega D, Coelho C, Vicente R, et al. AncesTrees: ancestry estimation with randomized decision trees. Int J Legal Med. 2015;129:1145–1153. 25053239 10.1007/s00414-014-1050-9

[38] Ousley SD. Forensic classification and biodistance in the 21st century: the rise of learning machines. In: Pilloud MA, Hefner JT, editors. Biological distance analysis: forensic and bioarchaeological perspectives. London (UK): Elsevier; 2016. p. 197–212.

[39] Purkait R. Triangle identified at the proximal end of femur: a new sex determinant. Forensic Sci Int. 2005;147:135–139. 15567617 10.1016/j.forsciint.2004.08.005

[40] Anastopoulou I, Eliopoulos C, Valakos ED, et al. Application of Purkait’s triangle method on a skeletal population from Southern Europe. Forensic Sci Int. 2014; 245:203.e1–203.e4. 25459271 10.1016/j.forsciint.2014.10.005

[41] Brown RP, Ubelaker DH, Schanfield MS. Evaluation of Purkait’s triangle method for determining sexual dimorphism. J Forensic Sci. 2007;52:553–556. 17456081 10.1111/j.1556-4029.2007.00423.x

[42] Djorojevic M, Roldán C, Botella M, et al. Estimation of Purkait’s triangle method and alternative models for sex assessment from the proximal femur in the Spanish population. Int J Legal Med. 2016;130:245–251. 25951948 10.1007/s00414-015-1201-7

[43] Eliopoulos C, Lagia A, Manolis S. A modern, documented human skeletal collection from Greece. HOMO. 2007;58:221–228. 17574249 10.1016/j.jchb.2006.10.003

[44] Herrera MD, Tallman SD. Craniometric variation and ancestry estimation in two contemporary Caribbean populations. Forensic Sci Int. 2019;305:110013. 31710881 10.1016/j.forsciint.2019.110013

[45] Stomfai S, Ahrens W, Bammann K, et al. Intra- and inter-observer reliability in anthropometric measurements in children. Int J Obes. 2011;35:45–51. 10.1038/ijo.2011.34 21483422

[46] Buikstra JE, Ubelaker DH. Standards for data collection from human skeletal remains. Research Series No. 44. Fayetteville: Arkansas Archeological Survey;1994.

[47] Miller EH, Mahoney SP, Kennedy ML, et al. Variation, sexual dimorphism, and allometry in molar size of the black bear. J Mammal. 2009;90:491–503.

[48] Albanese J, Eklics G, Tuck A. A metric method for sex determination using the proximal femur and fragmentary hipbone. J Forensic Sci. 2008;53:1283–1288. 18717754 10.1111/j.1556-4029.2008.00855.x

[49] Douglas AJ. A generalization of Apollonius’ theorem. Math Gaz. 1981;65:19–22.

[50] Darroch JN, Mosimann JE. Canonical and principal components of shape. Biometrika. 1985;72:241–252.

[51] Pedregosa F, Varoquaux G, Gramfort A, et al. Scikit-learn: machine learning in python. J Machine Learn Res. 2011;12:2825–2830.

[52] Python Software Foundation. [cited 2019 Dec 5]. Available from: http://python.org

[53] Hastie T, Tibshirani R, Friedman J. The elements of statistical learning: data mining, inference, and prediction. New York (NY): Springer; 2009.

[54] Strobl C, Boulesteix A, Zeileis A, et al. Bias in random forest variable importance measures: illustrations, sources and a solution. BMC Bioinform. 2007;8:25. 10.1186/1471-2105-8-25 PMC1796903 17254353

[55] Wang J. Random projection. In: Wang J, editor. Geometric structure of high-dimensional data and dimensionality reduction. Berlin (Germany): Springer; 2012. p. 131–148.

[56] Meshbane A, Morris JD. Assuming equal *vs.* unequal prior probabilities of group membership in discriminant analysis: effect on predictive accuracy. Paper presented at: the Annual Meeting of the American Educational Research Association; 1995; San Francisco, CA. Available from: https://files.eric.ed.gov/fulltext/ED392843.pdf

[57] Curate F, Coelho J, Gonçalves D, et al. A method for sex estimation using the proximal femur. Forensic Sci Int. 2016;266:579.e1–579.e7. 27373600 10.1016/j.forsciint.2016.06.011

[58] Lottering N, Reynolds MS, MacGregor DM, et al. Morphometric modelling of ageing in the human pubic symphysis: sexual dimorphism in an Australian population. Forensic Sci Int. 2014;236:e1–e11. 24468016 10.1016/j.forsciint.2013.12.041

[59] Cunha E, Ubelaker DH. Evaluation of ancestry from human skeletal remains: a concise review. Forensic Sci Res. 2020;5:89–97. doi:10.1080/20961790.2019.1697060 PMC7476619 32939424

[60] Colman KL, Dobbe JG, Stull KE, et al. The geometrical precision of virtual bone models derived from clinical computed tomography data for forensic anthropology. Int J Legal Med. 2017;131:1155–1163. 28185072 10.1007/s00414-017-1548-z PMC5491564

[61] Ubelaker DH, Ross AH, Graver SM. Application of forensic discriminant functions to a Spanish cranial sample. Forensic Sci Commun. 2002;4:1-6. https://archives.fbi.gov/archives/about-us/lab/forensic-science-communications/fsc/july2002/ubelaker1.htm

[62] Saunders SR, Hoppa RD. Sex allocation from long bone measurements using logistic regression. Can Soc Forens Sci J. 1997;30:49–60.

[63] Ousley S, Jantz R, Freid D. Understanding race and human variation: why forensic anthropologists are good at identifying race. Am J Phys Anthropol. 2009;139:68–76. 19226647 10.1002/ajpa.21006

[64] Witherspoon DJ, Wooding S, Rogers AR, et al. Genetic similarities within and between human populations. Genetics. 2007;176:351–359. 17339205 10.1534/genetics.106.067355 PMC1893020

[65] Mosimann JE. Multivariate analysis of size and shape: modelling with the Dirichlet distribution. Computer Science and Statistics: Proceedings of 19th Symposium on the Interface. Philadelphia, PA; 1987. p. 1–9.

[66] Jungers WL, Falsetti AB, Wall CE. Shape, relative size, and size-adjustments in morphometrics. Am J Phys Anthropol. 1995;38:137–161.

[67] Klingenberg CP. Size, shape, and form: concepts of allometry in geometric morphometrics. Dev Genes Evol. 2016;226:113–137. 27038023 10.1007/s00427-016-0539-2 PMC4896994

[68] Vercellotti G, Stout SD, Boano R, et al. Intrapopulation variation in stature and body proportions: social status and sex differences in an Italian medieval population (Trino Vercellese, VC). Am J Phys Anthropol. 2011;145:203–214. 21312185 10.1002/ajpa.21486

[69] Schlecht SH. Understanding entheses: bridging the gap between clinical and anthropological perspectives. Anat Rec (Hoboken). 2012;295:1239–1251. 22678817 10.1002/ar.22516

[70] Komar D. Identifying racial affinities in skeletal remains: utilizing infracranial non-metric traits and the Rubison procedure to determine racial identity. Can Soc Forens Sci J. 1996;29:155–164.

[71] Richman EA, Michel ME, Schulter-Ellis FP, et al. Determination of sex by discriminant function analysis of postcranial skeletal measurements. J Forensic Sci. 1979;24:159–167. 512599

[72] Anderson JY, Trinkaus E. Patterns of sexual, bilateral and interpopulational variation in human ­femoral neck–shaft angles. J Anatomy. 1998;192:279–285. 10.1046/j.1469-7580.1998.19220279.x PMC1467761 9643428

[73] Alunni V, Du Jardin P, Nogueira L, et al. Comparing discriminant analysis and neural network for the determination of sex using femur head measurements. Forensic Sci Int. 2015;253:81–87. 26093772 10.1016/j.forsciint.2015.05.023

[74] Hefner JT, Ousley SD. Statistical classification methods for estimating ancestry using morphoscopic traits. J Forensic Sci. 2014;59:883–890. 24646108 10.1111/1556-4029.12421

[75] Swamynathan M. Step 4: model diagnosis and tuning. In: Mastering machine learning with python in six steps. Berkeley (CA): Apress; 2019. p. 263–323.

[76] Shirley NR, Fatah EEA, Mahfouz M, et al. Beyond the cranium: ancestry estimation from the lower limb. In: Berg GE, Ta’ala SC, editors. Biological affinity in forensic identification of human skeletal remains: beyond Black and White. Boca Raton (FL): CRC Press; 2014. p. 133–153.

[77] Hefner JT, Spradley MK. Ancestry (forensic applications). In: Trevathan W, Cartmill M, Dufour D, et al., editors. The international encyclopedia of biological anthropology. New York (NY): John Wiley & Sons Inc.; 2018. p. 1–3.

[78] Bonneau N, Libourel P-A, Simonis C, et al. A three-dimensional axis for the study of femoral neck orientation. J Anatomy. 2012;221:465–476. 10.1111/j.1469-7580.2012.01565.x PMC3482355 22967192

[79] Rouge D, Telmon N, Arrue P, et al. Radiographic identification of human remains through deformities and anomalies of post-cranial bones: a report of two cases. J Forensic Sci. 1993;38:997–1007. 8355017

[80] Ubelaker DH. Human skeletal remains, excavation, analysis, interpretation. 3rd ed. Washington, DC: Taraxacum; 1999.

[81] Malakooti A. Migration trends across the Mediterranean. Interaction. 2016;44:16–21.

